# Efficacy of a Mobile Phone–Based Life-Skills Training Program for Addiction Prevention Optimized Among Adolescents With Low Program Engagement: Cluster-Randomized Controlled Trial

**DOI:** 10.2196/78081

**Published:** 2026-07-15

**Authors:** Severin Haug, Nikolaos Boumparis, Olivia Studhalter, Andreas Wenger, Michael P Schaub, Nikolai Kiselev

**Affiliations:** 1 Swiss Research Institute for Public Health and Addiction ISGF at University of Zurich, Konradstrasse 32, Zurich, 8005, Switzerland, 41 0444481174

**Keywords:** life skills, substance use, prevention, tailoring, adolescents, mobile phone

## Abstract

**Background:**

Previous studies demonstrated the effectiveness of *SmartCoach*, a mobile phone–based life-skills training program for addiction prevention among adolescents. However, socially stratifying factors, such as educational level or migration background, were associated with lower program engagement and participation. To address these disparities, we optimized and tailored program elements, particularly for subgroups with low engagement, using qualitative interview data.

**Objective:**

This study aimed to test whether the optimized program version was superior to the original one in terms of efficacy and program use. We hypothesized that optimization and advanced tailoring would result in increased program efficacy and engagement.

**Methods:**

A 2-arm, parallel-group, double-blind, cluster-randomized controlled trial was conducted with assessments at baseline and follow-up after 6 months. Secondary and upper secondary school classes were randomized into two groups: (1) an intervention group receiving the optimized program version and (2) a control group receiving the original version. Participants in both groups received up to 4 weekly SMS text messages over 4 months to stimulate (1) self-management skills to cope with stress, (2) self-efficacy to resist social pressure, (3) observational learning, and (4) self-regulation. The optimized program version included additional reminders, extended time for challenges, simplified language, different days for weekly messages, and additional tailoring for participants with personal or parental backgrounds from non–German-speaking countries. This subgroup received shorter video clips and information on additional stressors, including acculturation and family conflicts. The primary outcomes of this trial were the quantity of alcohol use, the number of days per month that nicotine-containing products were smoked, and cannabis use days.

**Results:**

A total of 1175 students from 67 Swiss secondary and upper secondary school classes were invited to participate in the study between September 2023 and September 2024. Of these 1175 students, 890 (75.7%; mean age 14.9, SD 1.3 y; women: n=511, 57.4%; men: n=379, 42.6%; nonbinary: n=0, 0.0%) participated in the study. Six-month follow-up assessments were completed by 552 (62.0%) of the 890 study participants. On average, program use did not differ between the study groups, with 12.6 (SD 12.8) of 38 possible activities completed in the intervention group and 12.5 (SD 12.7) activities in the control group (*P*=.18). The results concerning efficacy showed no significant differences between the study groups regarding the main outcomes of alcohol use quantity (*P*=.91), nicotine use days (*P*=.45), and cannabis use days (*P*=.67). Similarly, no significant group differences were observed for changes in perceived stress (*P*=.77) and social skills (*P*=.45).

**Conclusions:**

The results did not show improved program use or efficacy in the optimized version of the program. Possible explanations include the relatively minor differences between the 2 program versions and the generally low prevalence rates of substance use in this age group, which limited the statistical power of the study.

## Introduction

### Background

Substance use remains a significant public health issue in Western Europe and Switzerland [1]. This is particularly concerning due to the developmental vulnerability of adolescents, who are at a critical stage of brain maturation, particularly in areas related to impulse control, decision-making, and emotional regulation, which makes them more susceptible to risk-taking behaviors, including experimentation with substances [2,3]. Social and environmental influences also play a significant role. Adolescents are highly influenced by peer norms and social acceptance, with peer pressure and group dynamics often encouraging substance use [4,5]. Numerous meta-analyses and systematic reviews have assessed the effectiveness of school-based programs in preventing alcohol, tobacco, and other substance use [6-8]. Despite varying methodological rigor and theoretical foundations, these reviews highlight that school-based prevention can be effective, particularly when programs are grounded in psychosocial theory, build drug resistance and general competence skills, and are delivered interactively by teachers or program providers. Such life-skills intervention programs to prevent substance use [9-11] primarily combine training in self-management skills (eg, adapting to stress, emotional self-regulation, and goal setting), social skills (eg, assertiveness and communication skills), and skills facilitating resistance to substance use (eg, opposing peer pressure to drink alcohol and identifying and resisting media influences). However, their implementation and dissemination in schools present serious challenges [12,13]. First, teachers and other professionals need the time, motivation, knowledge, and skills to deliver the program. Second, extensive resources, in terms of personnel, money, and time allocated to deliver substance use prevention, are required to prepare and administer such programs [14].

Digital interventions have great potential to overcome the aforementioned obstacles that hinder successful program implementation and larger-scale dissemination of life-skills training in schools. A hybrid life-skills training program, combining e-learning modules and in-person sessions, among US middle school students, led to significant reductions in substance use and increases in health knowledge, skills knowledge, and life skills compared to a control condition [12].

*SmartCoach* (Swiss Research Institute for Public Health and Addiction) presented the first automated mobile phone–based life-skills training program to prevent substance use among adolescents, whose long-term effectiveness has been tested in a controlled study [15]. It includes online feedback and individually tailored SMS text messages provided over several months. The content is based on social cognitive theory and addresses self-management skills, social skills, and substance use resistance skills. The results on its efficacy at the 18-month follow-up showed that compared to controls, those in the intervention group experienced significantly lower tobacco smoking and cannabis use prevalence, while no effects were observed for alcohol use, well-being, or social skills [16].

A major challenge in delivering life-skills training and other preventive interventions electronically is the lack of control over adolescents’ engagement with the intervention. Several reviews on digital interventions to promote mental health [17,18] or to prevent substance use [19] in young people point to the relatively low levels of user engagement.

Poor program engagement typically jeopardizes program efficacy [20,21]. The *SmartCoach* efficacy study also showed several associations between engagement and intended outcomes, with adolescents who used the contests more frequently being more likely to be nonsmokers, and those who read the messages more attentively being less likely to drink in a problematic manner at the 6-month follow-up [22].

Several studies among adolescents and adults show associations between engagement in mobile health interventions and sociodemographic measures [22-24]. For example, in a SMS text messaging–based smoking cessation program in Switzerland, adolescents without an immigrant background showed higher program engagement [23]. In the *SmartCoach* program, students who attended a secondary school, as opposed to an upper secondary school, and those who reported a 2-sided immigration background (ie, both parents were not born in Switzerland), as opposed to those with no immigration background or a 1-sided immigration background, showed lower program engagement [22].

The challenge of low program use and engagement in mobile health (mHealth) interventions is increasingly discussed from scientific and practical perspectives; however, there is a lack of generalizable approaches that demonstrate how program engagement in existing mHealth programs can be systematically improved [25]. Promising approaches include advanced tailoring of intervention content [24,26] and involving adolescents in the design process to ensure the content is relevant and appealing to them [27,28].

These methods also provided the foundation for developing an optimized version of the *SmartCoach* program, which was tested in this study: first, subgroups with low program engagement were identified, and second, factors influencing user engagement and suggestions for program optimization were derived by using a participatory approach.

To identify subgroups with low program engagement, we evaluated several demographic and socioeconomic predictors of program participation and program engagement among 476 secondary and upper secondary school students in Switzerland in a previous study [29]. The results of that study concerning program engagement highlighted that a migration background, or more specifically, an origin from a non–German-speaking country, was the strongest single predictor of low program engagement [29].

Additionally, aiming to identify factors influencing user engagement and suggestions for the optimization of the *SmartCoach*, semistructured phone interviews were conducted among 171 program participants from the previous study [29]. The Capability, Opportunity, and Motivation model of behavior change [30] and the theoretical domains framework (TDF) [31] were used to assess behavioral influences [32].

Next to the general recommendations for advanced tailoring of the *SmartCoach* program content, specific recommendations for the subgroup of students originating from non–German-speaking countries were also derived from the previous qualitative study and implemented in this study (see the Methods section) [32].

### Study objectives

The main objective of this study was to test program engagement and the efficacy of the *SmartCoach* version optimized in this way, compared to the original program version without advanced tailoring. We hypothesized that receiving the optimized intervention program, compared to the original version, would result in increased program efficacy, indicated by lower substance use at 6-month follow-up (primary outcomes), increased program engagement and social skills, as well as decreased levels of stress (secondary outcomes).

## Methods

### Study Design

This study aimed to test program engagement and the efficacy of the optimized program version with advanced tailoring compared to the original program version in a 2-arm parallel-group, double-blind, randomized controlled trial with assessments at baseline and at a 6-month follow-up.

### Participants, Setting, and Procedure

In this study, secondary and upper secondary schools in the Swiss cantons of Aargau and Zurich were invited to participate by prevention experts from regional addiction prevention centers. Interested teachers were informed about the program’s objectives and its implementation in their classes, and they allocated 45 to 90 minutes during regular school lessons for the program.

During this time, students were introduced to the topic through a brief workshop, informed about the program and the accompanying study, and invited to participate and sign up for the study. The workshop and information session were led by prevention experts or psychology master’s students experienced in working with young people and delivering preventive interventions.

The 30‐ to 60-minute workshop aimed to spark students’ interest in stress and provide basic information on its origins. Initially, students shared their understanding of stress in a group discussion. The concept of stress as an imbalance between demands and resources was then explained. Interactive exercises, such as repeatedly subtracting 7 from 996, were used to deliberately induce stress, allowing students to observe its effects on their bodies and thoughts. Finally, students described their stress levels in areas like family, friends, school, and the future using emojis.

Following this, students were introduced to the *SmartCoach* program and the related study through an introductory video [33]. Students were invited to participate in the study if they met the following criteria: (1) were at least 14 years old and (2) owned a mobile phone.

They were asked to complete an online baseline assessment and study registration using their smartphones. Students who consented to participate provided a self-defined username and mobile phone number and completed additional assessments on stress and social skills for tailoring the intervention content. They received individually tailored web-based feedback on their mobile phones. Students who did not give consent were thanked for their interest and asked to explain their reasons for not participating in a free-text field.

Those who decided to participate received personalized mobile phone–based life-skills training over the next 4 months. Follow-up assessments at month 6 in both study groups were conducted using a similar procedure: participants were invited to the online follow-up assessments via SMS text messaging, which included a link to the follow-up survey. After 3 reminders, nonresponders were additionally contacted via computer-assisted telephone interviews conducted by research assistants. Study participants were recruited between September 2023 and September 2024. The 6-month follow-up assessments were conducted between March 2024 and April 2025.

### Ethical Considerations

The study was approved by the Ethics Committee of the Faculty of Arts and Sciences at the University of Zurich (approval 22.2.15; date of approval April 17, 2022). Digital informed consent was obtained from all study participants. Parental consent was required only for children under 15 years of age because the study was classified by the ethics committee as a research project with minimal risk [34]. Study participants completed the screening procedure and baseline assessment anonymously. No names were collected; instead, participants chose nicknames. Password protection and secure sockets layer encoding were used to ensure the privacy and security of data transfer. Study participants received an incentive of 10 Swiss Francs (1 Swiss Franc=US $1.23 as of June 23, 2026) for taking part in the follow-up survey.

### Randomization and Allocation Concealment

To prevent spillover effects within school classes, we conducted a cluster-randomized controlled trial, using school classes as the randomization unit. Multiple classes per school were randomized. Given the diverse student populations across different secondary schools, we used stratified randomization with separate computer-generated randomization lists for each school. In addition, to ensure balanced sample sizes across study groups, we used block randomization with computer-generated randomly permuted blocks of 4 cases [35].

Prevention experts and master’s students conducting the baseline assessment were blinded to the group allocation of school classes. Furthermore, the research assistants who performed the computer-assisted follow-up assessments for primary and secondary outcomes were also blinded to the group allocation. Additionally, students did not know whether they were allocated to the original or optimized programs.

### Intervention Program

#### Original Program Version

**Figure 1.**
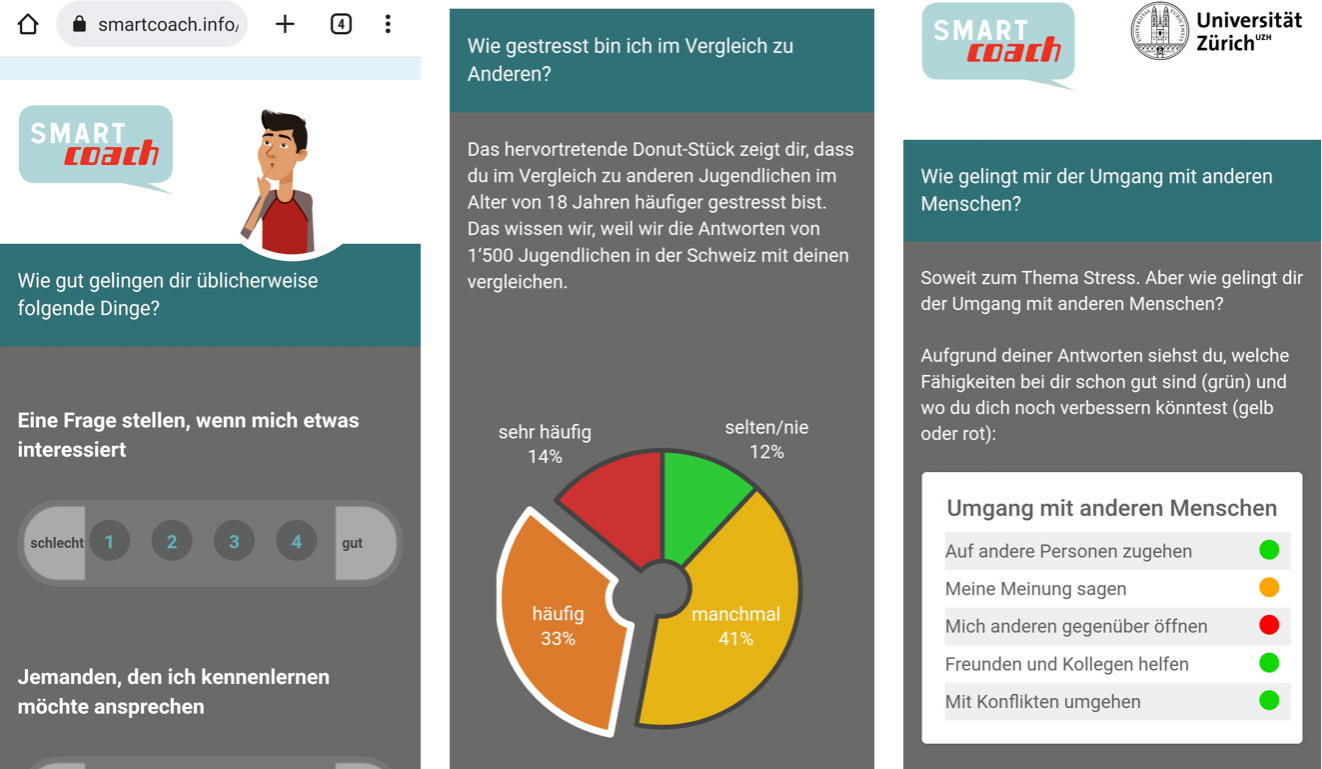
Screenshots from the *SmartCoach* program (left: assessment of social skills; middle: feedback on level of stress; right: feedback on individual social skills).

The intervention elements of the program were based on the social cognitive theory [36,37]. The key concepts of this theory, which were addressed within *SmartCoach,* were (1) outcome expectations, (2) self-efficacy, (3) observational learning, (4) facilitation, and (5) self-regulation. The *SmartCoach* program [33] provided tailored web-based feedback and SMS text messages to promote self-management skills, social skills, and substance use resistance skills over a period of 4 months. The intervention elements of the program were based on social cognitive theory [36,37] and the life-skills training [9]. Individually tailored web-based feedback was provided to program participants immediately after they completed the baseline assessment within their school classroom. This included textual and graphical feedback on general stress, levels of stress in various domains, individual applied and suggested coping strategies, and individual levels of social skills. Screenshots of the baseline assessment and the web-based feedback are shown in Figure 1.

Following the baseline assessment and feedback, program participants received between 2 and 4 individualized SMS text messages per week on their mobile phones for a period of 17 weeks. These messages were generated and sent by a fully automated (not artificial intelligence–based) system. For the first 7 weeks, the messages focused on self-management skills, for example, coping with stress, emotional self-regulation, and managing anger and frustration. In weeks 8 to 12, the messages focused on social skills, for example, making requests, refusing unreasonable requests, and meeting new people. In weeks 13 to 17, the SMS text messages focused on substance use resistance skills, for example, recognizing and resisting media influences, social norms of alcohol and tobacco use, and the associations between both self-management and social skills and substance use. The messages were tailored based on data from the baseline assessment and on SMS text messaging assessments that occurred over the course of the program.

During the 4-month coaching phase, participants received a total of 38 SMS text message prompts that invited interaction, such as replying to quizzes, retrieving media objects, and participating in challenges or contests. A friendly competition among participants was integrated into the program to stimulate engagement. Within the friendly competition, program participants could collect credits for each interaction (eg, answering monitoring SMS text messages, participating in quizzes, creating messages or pictures within contests, and accessing video links integrated into SMS text messages). The more credits participants collected, the higher their chances were of winning one of several attractive prizes, which were part of a prize draw (10 prizes with a total value of 500 Swiss Francs, eg, cinema tickets) after program completion. Participants were able to retrieve their number of credits compared to the number of credits of other program participants in their group (with a similar starting date) at any time from an individual profile page. Sample screenshots of the program from the coaching phase are shown in Figure 1.

#### Optimized Program Version

The optimized program version was developed as described in the “Background” section based on a quantitative study that identified students from non–German-speaking countries as a subgroup with particularly low program engagement [29], as well as on a qualitative examination of suggestions for program optimization in this subgroup and also among other program participants [32]. The optimized program version included additional tailoring for participants with personal or parental backgrounds from non–German-speaking countries, who received shorter video clips. Furthermore, other stressors, including acculturation and family conflicts, were addressed, and the weekly SMS text messages were not always sent on Tuesdays but on different days of the week. A comprehensive overview of the program optimization version is displayed in Table 1.

**Table 1.** Program optimizations for all participants and additional optimizations for those with personal or parental backgrounds from non–German-speaking countries.

Suggestions from qualitative study [32]	Optimizations for all participants	Additional optimizations for participants with personal or parental backgrounds from non–German-speaking countries
Shorter videos	—^a^	Videos shortened to a maximum duration of 2 min.
Simplifying language	Complex or long sentences were revised and simplified to ensure clarity and accessibility.	—
General reminders	Participants who did not respond to certain prompts received additional reminder messages to encourage engagement.	—
Additional reminder message for upload within group challenges	Participants received a reminder message 1 h before the end of the group challenge submission deadline if no action was taken.	—
Reminder for voting in self-challenges	Participants received a reminder 1 h before the voting phase ended if they had not yet voted.	—
SMS on different days	Weekly interactive messages (eg, quizzes) were sent on 2 different days (Tuesdays and Fridays) rather than always on Tuesdays to better fit diverse routines.	—
Adapted timing for challenge participation	The time window for submitting entries to group challenges was adjusted to earlier in the afternoon (and prolonged from 2-4 h this way) to accommodate daily schedules.	—
Adapted timing for voting on challenges	The voting period was extended until 3 PM (an additional 3 h), allowing more flexibility for participants to engage.	—
Time for publication of group challenge results	Challenge results were sent out shortly after the extended voting period ended.	—
Regular updates on personal progress	Additional updates about collected points and status in the friendly competition were sent periodically.	—
Ensuring anonymity in group interactions	The program provided clearer and simplified explanations about anonymity to increase comfort and trust in participation.	—
More differentiated assessment of stress factors	The stress assessment was refined to distinguish between stress from family conflicts and stress from peer conflicts.	The explanation of family conflict stress included examples relating to cultural differences and intergenerational misunderstandings.
Addressing feelings of exclusion	—	New content was added to reflect on feelings of being an “outsider,” including migration-related themes like acculturation and identity development.
Addressing future-related stress	—	Specific questions and feedback regarding worries about the future were tailored to migration-related challenges (eg, education and residence status).

a Not applicable.

### Assessments and Outcomes

#### Sociodemographic Data

Assessments of demographic and socioeconomic variables included the birth country of both parents and the students to determine a migration background. Since Swiss-German people are similar to Germans and Austrians in terms of language, culture, and socioeconomic background, a further variable was created based on the students’ and their parents’ country of birth, which describes their origin from a non–German-speaking country. Furthermore, we assessed gender, age, and perceived familial socioeconomic status from the perspective of the adolescent using a brief instrument [38,39] that was translated, adapted, and validated for German adolescents [40].

#### Program Use

Program use was operationalized in terms of the total number of interactions with the program that were logged by the system. During the 4-month coaching phase, participants received a total of 38 SMS text messages that prompted a total of 18 SMS text messaging–based reply activities (eg, replies to quiz questions or selection queries) and 20 media view activities (eg, views of video clips, images, or weblinks). These activities were logged through the *SmartCoach* system and were available for each program participant.

#### Outcomes

Baseline and follow-up assessments included the following:

- Alcohol use in the preceding 30 days was assessed by the consumption items of the Alcohol Use Disorders Identification Test (AUDIT-C) [41]. This test comprises three items assessing (1) frequency of alcohol consumption, (2) quantity of alcohol consumption, and (3) binge drinking, and it was also validated for use among adolescents [42]. Pictures were used to illustrate the quantity of a standard drink, which corresponds to 12 to 14 g of pure alcohol.
- Nicotine or tobacco use was assessed using the item: “Have you smoked a puff of nicotine-containing cigarettes, e-cigarettes, or vapes within the last 30 days?” If the participant responded “yes,” we additionally assessed the number of nicotine or tobacco use days in the past 30 days.
- Cannabis use in the preceding 30 days was assessed using the item: “Have you smoked or used products containing cannabis within the last 30 days?” If the participant responded “yes,” we additionally assessed the number of days they used cannabis.
- Perceived stress was assessed by a single item from the Swiss Juvenir study [43]: “How often have you had the feeling of being overstressed or overwhelmed in the last month?” with the answer options ranging from 1 “never” to 5 “all the time.”
- Social skills were assessed using the brief version of the Interpersonal Competence Questionnaire-10 [44], which addresses the following domains: initiation of relationships, negative assertion, disclosure of personal information, emotional support, and conflict management.

The primary outcomes, according to the study registration, were alcohol use in the preceding 30 days, as measured by the AUDIT-C total score; the number of nicotine or tobacco use days in the past 30 days; and the number of cannabis use days in the preceding 30 days. Secondary outcomes included perceived stress, social skills, and program use captured via the total number of interactions with the program that were logged by the system.

### Sample Size

The effect size calculations for this trial were based on a meta-analysis of the efficacy of SMS text messaging–based interventions for health promotion [26]. It tested a variety of participant, intervention, and methodological moderators and identified message tailoring, as well as personalization, as being significantly associated with greater intervention efficacy. The largest effect sizes were exhibited in those studies that used both message tailoring and targeting (Cohen *d*=0.44), followed by studies using message tailoring only (Cohen *d*=0.27), whereas the smallest effects were observed among studies that used only message targeting (Cohen *d*=0.07). Furthermore, the analysis also examined the impact of tailoring SMS text messages specifically based on demographic factors in message design. Interventions were significantly more efficacious when they used demographic factors (Cohen *d*=0.53) in tailoring than when they did not (Cohen *d*=0.22). Based on these findings, we expected an increased effectiveness of the optimized program version, which provides advanced tailoring based on several socially stratifying factors, by a small to medium magnitude. Assuming a Cohen *d* of 0.25, a sample size of 253 in each study group would be required to achieve 80% power for a *t* test (*α*=5%, 2-sided) to detect this difference based on a calculation using G*Power (Heinrich Heine University Düsseldorf). As secondary school students are nested within school classes, we additionally needed to consider a potential design effect for the calculation of the sample size for our study. Based on an average cluster size of 15 study participants [16] and an expected intracluster correlation coefficient of 0.05, a sample size of 430 per study group and a total of 860 study participants was required.

### Data Analysis

To examine baseline differences between participants in the intervention and control groups, we performed chi-square tests for categorical variables, *t*-tests, and Mann-Whitney *U* tests for continuous variables. The same tests were applied to assess whether participants lost to follow-up differed from those who responded as a function of the study group.

We analyzed data according to the intention-to-treat principle (ITT). For the ITT analyses, we used multiple imputation procedures as described elsewhere [45]. Specifically, the imputation model included study group, demographic variables (gender, age, immigration background, and socioeconomic status), school-related factors (school type and class), baseline measures of all outcomes (stress, social skills, and substance use), and program engagement metrics. We generated 20 imputed datasets with 5 iterations each, and no systematic bias in convergence was observed. Binary variables were imputed using logistic regression, and both count and continuous variables were imputed using predictive mean matching.

For all outcomes, we used generalized linear mixed models (GLMMs) with a random intercept for the school class. For count outcomes, we used Poisson GLMMs with a log link function and included the log of the baseline measure plus one as an offset term to model relative changes. For dichotomous outcomes, we used binomial GLMMs with a logit link. For continuous outcomes, we used linear mixed models with change scores (follow-up minus baseline) as the dependent variable. All models included the study group as the primary predictor, with age, gender, school type, juvenile stress, and baseline cannabis use as covariates. We included similar covariates in the program engagement models.

We did not adjust for multiple comparisons, as this was not prespecified in the sample size calculation or study protocol. To avoid post hoc bias, analyses followed the original protocol, using predefined significance thresholds without multiplicity correction.

To identify the moderators of the primary and secondary intervention outcomes, GLMMs with a random intercept for school class were used. Interaction terms between sociodemographic characteristics (gender, migration status, origin from a non–German-speaking country, socioeconomic status) measured at baseline and study group were included, one at a time, in the previously described models.

Results with a type I error rate of *P*<.05 on 2-sided tests were considered statistically significant. Analyses were performed using R version 3.6.1 with the lme4 package [45] for fitting mixed models.

## Results

### Study Participants

**Figure 2.**
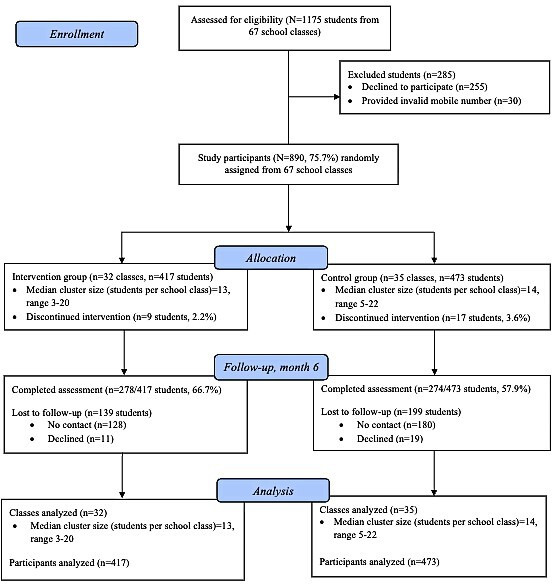
Flowchart of study participants.

Figure 2 depicts participants’ progression through the trial. At the online screening assessment, 1175 students were present in 67 classes. Of these 1175 students, 890 (75.7%) consented to participate in the study and provided a valid mobile phone number. Of the 255 students who declined to participate, 83 (32.5%) provided usable free-text information about their reasons for not taking part in the program and associated study. The most common reason given was a lack of desire or interest in participating in the program (n=47, 57%). A total of 22 (27%) students stated that they did not have their smartphone with them; another 5 (6%) indicated that they did not have a smartphone at all. Three (4%) students said they were unwell. Two (2%) students mentioned privacy concerns, 2 (2%) did not have parental consent, and 2 (2%) experienced technical difficulties with the internet or their smartphones.

Thirty-two classes containing 417 students in total were randomly assigned to the intervention group, and 35 classes with 473 students in total were assigned to the control group. Six-month follow-up assessments were completed by 278 of 417 (66.7%) students in the intervention groups and 274 of 473 (57.9%) students in the control group.

Baseline characteristics for the study sample are shown in Table 2. The mean age was 14.9 (SD 1.3) years, and 511 (57.4%) of the 890 study participants were women.

Baseline differences between the intervention and control groups were identified for age, type of school, and perceived stress.

Concerning attrition bias, the analysis revealed that intervention group participants who were lost to follow-up were more likely to be men (*χ*²_1_=8.5; *P*=.003) and had a higher cannabis use prevalence at baseline (*χ*²_1_=6.0; *P*=.014). Furthermore, intervention group participants lost to follow-up were more likely to have lower levels of perceived stress (*F*_1,336_=4.8; *P*=.03) than controls.

The intracluster correlation coefficients (ICCs) for the 3 primary outcomes were as follows: for AUDIT-C, ICC of 0.12 at baseline and 0.03 at follow-up and for nicotine or tobacco use days, ICC of 0.08 at baseline and 0.03 at follow-up. For cannabis use days, the random-intercept models did not converge at baseline and follow-up, indicating negligible between-cluster variance.

**Table 2.** Baseline characteristics of the study sample.

Variable	Intervention (n=417)	Control (n=473)	Total (N=890)	*P* value^a^
Gender, n (%)				.42^b^
Man	184 (44.1)	195 (41.2)	379 (42.6)	
Woman	233 (55.9)	278 (58.8)	511 (57.4)	
Nonbinary or other	0 (0)	0 (0)	0 (0)	
Age (y), mean (SD)	15.2 (1.3)	14.7 (1.3)	14.9 (1.3)	<.001^c^
Migration background, n (%)				.29^b^
No	133 (31.9)	167 (35.3)	300 (33.7)	
Yes	284 (68.1)	306 (64.7)	590 (66.3)	
Origin from a non–German-speaking country, n (%)				.31^b^
No	166 (39.8)	205 (43.3)	371 (41.7)	
Yes	251 (60.2)	268 (56.7)	519 (58.3)	
Type of school, n (%)				.004^b^
Secondary school	264 (63.3)	343 (72.5)	607 (68.2)	
Upper secondary school	153 (36.7)	130 (27.5)	283 (31.8)	
Socioeconomic status, mean (SD)	6.6 (1.6)	6.5 (1.5)	6.6 (1.5)	.57^c^
Perceived stress, mean (SD)	3.2 (1.0)	3.4 (1.0)	3.3 (1.0)	.004^c^
Social skills (ICQ-10^d^), mean (SD)	28.6 (4.8)	28.3 (4.7)	28.4 (4.8)	.35^c^
Nicotine or tobacco use in preceding 30 d, n (%)				.31^b^
No	329 (78.9)	387 (81.8)	716 (80.4)	
Yes	88 (21.1)	86 (18.2)	174 (19.6)	
Nicotine or tobacco use days in preceding 30 d, mean (SD)	3.0 (8.0)	2.4 (7.1)	2.6 (7.5)	.23^e^
Alcohol use, preceding 30 d (AUDIT-C score^f^, range 0‐12), mean (SD)	1.2 (2.4)	1.1 (2.2)	1.1 (2.3)	.32^e^
Cannabis use, preceding 30 d, n (%)				.09^b^
No	386 (92.6)	451 (95.3)	837 (94.0)	
Yes	31 (7.4)	22 (4.7)	53 (6.0)	
Cannabis use days, preceding 30 d, mean (SD)	0.5 (3.2)	0.3 (2.0)	0.41 (2.6)	.08^e^

a *P* values for the comparison of the intervention and control groups.

b Chi-square test.

c *t* test.

d ICQ-10: Interpersonal Competence Questionnaire-10.

e Mann-Whitney *U* test.

f AUDIT-C: consumption items of the Alcohol Use Disorders Identification Test.

### Program Use and Engagement

During the intervention program, which lasted for 4 months (17 wk), 9 (2.2%) of the 417 program participants in the intervention group and 17 (3.6%) of the 473 participants of the control group withdrew their participation (odds ratio 0.50, 95% CI 0.08‐3.15; *P*=.46). Over the 4-month program, a total of 18 SMS text messaging–based reply activities (eg, replies to quiz questions or selection queries) and 20 media view activities (eg, views of video clips or images) were prompted. Considering the total number of 38 possible activities, the mean number of activities carried out by participants was 12.6 (SD 12.8) in the intervention group and 12.5 (SD 12.7) in the control group (unstandardized regression coefficient from the linear mixed model *b*=−1.72, 95% CI −4.22 to 0.79; *P*=.18). Considering the number of SMS text messaging replies and media view activities separately, program engagement also did not differ significantly between the study groups: a mean number of 7.2 (SD 6.8) SMS text message reply activities were completed in the intervention group and 7.3 (SD 6.8) in the control group (*b*=−1.12, 95% CI −2.43 to 0.18; *P*=.10), while a mean of 5.4 (SD 6.6) media view activities were completed in the intervention group and 5.2 (SD 6.6) in the control group (*b*=0.18, 95% CI −0.92 to 1.28; *P*=.74).

### Efficacy Based on 6-Month Follow-Up

The results of the complete-case (CC) and ITT analyses examining outcomes at the 6-month follow-up are presented in Tables3  4. For count variables (Table 3), no significant group differences were observed for tobacco use days (ITT: rate ratio=0.75; *P*=.45) or cannabis use days (ITT: rate ratio=0.86; *P*=.67) in either the CC or ITT analyses.

Continuous outcomes (Table 4) including AUDIT-C score (ITT: *P*=0.91), perceived stress (ITT: *P*=0.77), and social skills (ITT: *P*=0.45) also did not differ significantly between groups in either CC or ITT analyses.

**Table 3.** Intervention effects for count outcomes.

Outcome	Intervention group (n=417)			Control group (n=473)				*P* value
	Baseline	Follow-up	Difference	Baseline	Follow-up	Difference	Rate ratio (95% CI)	
Complete-case analysis								
Nicotine or tobacco use days in preceding 30 d, mean (SD)	3.0 (8.0)	2.1 (6.8)	−0.9	2.4 (7.1)	2.4 (7.2)	0.0	0.66 (0.31-1.43)	.29
Cannabis use days, preceding 30 d*,* mean (SD)	0.5 (3.2)	0.2 (1.4)	−0.3	0.3 (2.0)	0.5 (3.1)	0.2	0.41 (0.11-1.47)	.17
Intention-to-treat analysis								
Nicotine or tobacco use days in preceding 30 d, mean (SD)	3.0 (8.0)	4.0 (8.7)	1.0	2.4 (7.1)	4.6 (9)	2.2	0.75 (0.36-1.57)	.45
Cannabis use days, preceding 30 d*,* mean (SD)	0.5 (3.2)	1.7 (4.5)	1.2	0.3 (2.0)	2.2 (5.2)	1.9	0.86 (0.43-1.73)	.67

**Table 4.** Intervention effects for continuous outcomes.

Outcome	Intervention group (n=417)			Control group (n=473)			Unstandardized regression coefficient *b* (95% CI)	*P* value
	Baseline	Follow-up	Difference	Baseline	Follow-up	Difference		
Complete-case analysis								
AUDIT-C^a^ score, mean (SD)	1.2 (2.4)	1.0 (1.9)	−0.2	1.1 (2.2)	0.8 (2.1)	−0.3	0.032 (−0.338 to 0.402)	.87
Perceived stress past 30 d, mean (SD)	3.2 (1.0)	2.8 (1.0)	−0.4	3.4 (1.0)	2.8 (1.0)	−0.6	−0.013 (−0.186 to 0.160)	.89
Social skills (ICQ-10^b^), mean (SD)	28.6 (4.8)	30.9 (4.4)	2.3	28.3 (4.7)	30.8 (4.6)	2.5	−0.101 (−1.285 to 1.083)	.87
Intention-to-treat analysis								
AUDIT-C score, mean (SD)	1.2 (2.4)	1.5 (2.4)	0.3	1.1 (2.2)	1.6 (2.6)	0.5	0.030 (−0.473 to 0.533)	.91
Perceived stress past 30 d, mean (SD)	3.2 (1.0)	2.7 (1.0)	−0.5	3.4 (1.0)	2.8 (1.0)	−0.6	−0.034 (−0.262 to 0.195)	.77
Social skills (ICQ-10), mean (SD)	28.6 (4.8)	30.9 (4.4)	2.3	28.3 (4.7)	30.8 (4.6)	2.5	−0.525 (−1.881 to −0.832)	.45

a AUDIT-C: consumption items of the Alcohol Use Disorders Identification Test.

b ICQ-10: Interpersonal Competence Questionnaire-10.

### Moderator Analyses

No significant demographic or socioeconomic variables moderated the effect of the intervention on the primary or secondary outcomes.

## Discussion

### Principal Results

This study evaluated program engagement and the efficacy of an optimized mobile phone–based life-skills training program with advanced tailoring of intervention content, compared to the original program version among adolescents. The results revealed no significant differences between participants of the 2 program versions with respect to the considered indicators of program engagement and efficacy.

The proactive invitation to participate in the program and study in Swiss secondary school classes, combined with the offer of a low-threshold mobile phone–based intervention, enabled us to reach 3 out of 4 adolescents for participation in the *SmartCoach* program and the associated study. Given that the program lasted 17 weeks and that participants had to provide their mobile phone numbers, the high participation rate of 75.7% (890/1175) is remarkable though consistent with previous studies of the program in this target group [16,29].

Concerning program attrition, the overall results were positive, with only 2.2% (9/417) of the participants in the intervention group and 3.6% (17/473) of the control group withdrawing their participation. Relating to program use, the average number of completed activities was 13 out of 38 possible activities in both study groups, with no significant between-group differences either in this total number or in the more specific activities of SMS text message replies or media views. The moderator analyses also showed no differences in the program participation variables. Therefore, the initial hypothesis that the program optimizations or the advanced tailoring of program content would result in increased program use has to be rejected. Similarly, no statistically significant group differences were observed for the primary and secondary outcomes reflecting program efficacy.

Despite previous evidence supporting the effectiveness of *SmartCoach*, the optimized version did not demonstrate significant improvements over the original program. One critical factor that may have contributed to these nonsignificant results is the lack of statistical power, which might be related to the generally low substance use prevalence rates in this age group that may not have been sufficient to detect the relatively small differences between the optimized and original program versions. In relation to the overall program content and processes, only a small proportion of the content (maximum 8%, depending on the topic choice during the assessment) was changed. This is also due to the fact that, in the qualitative program evaluations relating to the Capability, Opportunity, and Motivation model of behavior change and theoretical domains framework, most young people were very satisfied with the original program and its content, and there were relatively few requests for changes [32]. At the same time, it must be mentioned that this previous study did not reach the entire sample for the follow-up interviews, and some decisions to change program content were based on relatively small numbers. Furthermore, these specific adaptations might not have addressed the participants’ real challenges regarding program engagement or may even have had a negative impact (eg, reminders might be perceived as annoying and evoke reactance; messages on a different day of the week might be helpful for some but could also be counterproductive for another subgroup). Future studies in which optimization suggestions are assessed should therefore collect them in two steps: first, optimization suggestions should be identified qualitatively, and in a second step, an independent sample should be used to check quantitatively whether these suggestions are desired by the majority or whether they are only the preferences of a minority. Another reason for the zero effects may be that the assumed effects of advanced tailoring were overly optimistic. One reason for this is that the 3 project packages (identification of subgroups with low program engagement, qualitative exploration and implementation of program optimizations, and this randomized controlled trial to test the effectiveness of the optimized program) were planned together in advance, including power calculations. Initially, we assumed that, in addition to the variable “parental background from non–German-speaking countries,” other variables such as health literacy, socioeconomic status, and gender might influence program engagement. However, it turned out that they did not have a substantial effect in this initial study, and both the number of variables that could be used for improved tailoring and the necessary scope of program optimizations were rather small [29,32].

### Limitations

Main limitations of this study are that (1) all primary outcome data rely on self-report, with the associated possibility that results may have been influenced by social desirability and potential recall bias. Measures used to avoid under- or overreporting of substance use included assurances of confidentiality and anonymous assessments conducted via online surveys without personal contact, which may have increased the reliability of self-reported data. (2) Cluster randomization according to school class did not result in a balancing of all baseline characteristics, and there was selective attrition according to gender, cannabis use prevalence, and perceived stress. Although we attempted to counteract this attrition bias as effectively as possible through multiple imputation, ITT analyses, and additional complete case analyses for sensitivity testing, a certain degree of bias and its effect on the results cannot be completely ruled out. (3) The results could not be generalized to secondary and upper secondary schools in Switzerland, as we recruited a convenience sample of school classes willing to participate in the study. However, the comparison of the substance use prevalence rates among a representative sample of 15-year-old students in Switzerland [1] and the baseline characteristics of the study sample does not reveal major deviations. The 30-day point prevalence rates for nicotine use were 20% in this study and 16% for cigarette consumption, with an additional 3% for e-cigarette use in the representative survey. For cannabis use, the 30-day point prevalence was 6% in this study and 9% in the national survey.

### Conclusions and Outlook

This study is among the first to evaluate the impact of a mobile phone–based life-skills training program for adolescent substance use prevention, which integrates participatory tailoring based on user feedback within a randomized controlled trial setting. The results demonstrate that the program was feasible and acceptable, as indicated by a high participation rate and relatively low program attrition. However, the optimized version did not show improved engagement or efficacy compared to the original version.

Despite the lack of superiority of the optimized program version, the *SmartCoach* program offers a low-threshold and effective intervention program to prevent substance use among adolescents [16]. The SMS text messaging–based version is currently being revised as part of a European Union–funded project to create an instant messaging version using WhatsApp (Meta Platforms). This offers significant advantages over SMS, including the ability to send media such as pictures and video clips directly, as well as the option to send messages free of charge using an existing internet connection [46]. Furthermore, this instant messaging–based coaching program will integrate storytelling elements to provide meaning to the facts presented and allow participants to connect with the information both intellectually and emotionally [47]. For example, in the new program version, participants will accompany a Stone Age man or woman (Stoney) on a journey through a picture story to discover the origins of stress and learn how to cope with stressful situations. These situations will be based on those that participants provided information about during the baseline assessment. Compared to the revisions implemented in the context of this study, these more substantial program revisions could lead to better retention and engagement, ultimately improving the efficacy of the *SmartCoach* life-skills training program.

## Supplementary material

10.2196/78081 Checklist 1 CONSORT-eHEALTH checklist.

## Abbreviations

AUDIT-C: consumption items of the Alcohol Use Disorders Identification Test
CC: complete case
CONSORT: Consolidated Standards of Reporting Trials
GLMM: generalized linear mixed model
ICC: intracluster correlation coefficient
ITT: intention-to-treat
mHealth: mobile health
